# Immobilization of *Clostridium acetobutylicum* onto natural textiles and its fermentation properties

**DOI:** 10.1111/1751-7915.12557

**Published:** 2017-01-23

**Authors:** Wei Zhuang, Xiaojing Liu, Jing Yang, Jinglan Wu, Jingwei Zhou, Yong Chen, Dong Liu, Hanjie Ying

**Affiliations:** ^1^ State Key Laboratory of Materials‐Oriented Chemical EngineeringNanjing Tech UniversityNo. 5, Xinmofan RoadNanjing210009China; ^2^ College of Biotechnology and Pharmaceutical EngineeringNational Engineering Technique Research Center for BiotechnologyNanjing Tech UniversityNo. 30, Puzhu South RoadNanjing211816China; ^3^ Synergetic Innovation Center for Advanced MaterialsNanjing Tech UniversityNo. 30, Puzhu South RoadNanjing211816China

## Abstract

Immobilized fermentation has several advantages over traditional suspended fermentation, including simple and continuous operation, improved fermentation performance and reduced cost. Carrier is the most adjustable element among three elements of immobilized fermentation, including carrier, bacteria and environment. In this study, we characterized carrier roughness and surface properties of four types of natural fibres, including linen, cotton, bamboo fibre and silk, to assess their effects on cell immobilization, fermentation performance and stability. Linen with higher specific surface area and roughness could adsorb more bacteria during immobilized fermentation, thereby improving fermentation performance; thus, linen was selected as a suitable carrier and was applied for acetone–butanol–ethanol (ABE) fermentation. To further improve fermentation performance, we also found that microbes of *Clostridium acetobutylicum* were negatively charged surfaces during fermentation. Therefore, we then modified linen with polyetherimide (PEI) and steric acid (SA) to increase surface positive charge and improve surface property. During ABE fermentation, the adhesion between modified linen and bacteria was increased, adsorption was increased about twofold compared with that of unmodified linen, and butanol productivity was increased 8.16% and 6.80% with PEI‐ and SA‐modified linen as carriers respectively.

## Introduction

Currently, there are major concerns worldwide regarding pollution and the greenhouse effect, resulting from excessive use of fossil fuels (Peralta‐Yahya *et al*., 2012). Moreover, because of impending depletion of these nonrenewable sources, scientists around the globe are focusing on development of environmentally friendly, technologically feasible and renewable energy sources (Durre, 2007; Xue *et al*., 2013). Biomass energy (e.g. bioethanol, biodiesel and biobutanol) may represent a feasible alternative. For example, biobutanol produced by *Clostridium acetobutylicum* (*C. acetobutylicum*) is a valuable biofuel due to its high energy content, low volatility, reduced hygroscopicity and low corrosiveness (Qureshi and Maddox, 1987; Bankar *et al*., 2013). Moreover, biobutanol can be used directly by regular gasoline engines without modifications and/or substitutions and may have applications as an intermediate or solvent in the chemical and textile industries (Chen *et al*., 2013; Liu *et al*., 2014).

Biobutanol production is generally achieved through suspended fermentation processes. Notably, cell immobilization may be an attractive technique for biobutanol production and may have many advantages over conventional suspended fermentation, including higher cell densities (leading to higher productivity; Friedl *et al*., 1991; Survase *et al*., 2012), shorter fermentation time, ability to facilitate continuous processing, increased tolerance to high substrate concentration, reduced product inhibition, reduced risk of microbial contamination and reduced cost (Kourkoutas *et al*., 2004; Bucko *et al*., 2012; Dolejš *et al*., 2014). In cell‐immobilized fermentation, cells are physically confined or localized to a certain area to preserve some desired catalytic activity (Goulter *et al*., 2010; Choi *et al*., 2015). This process involves four steps: (i) attachment or adsorption, (ii) entrapment, (iii) aggregation by self or with cross‐linking agents, and (iv) cell containment behind barriers (Pilkington *et al*., 1998; Kourkoutas *et al*., 2004; Borovikova *et al*., 2014; Zhuang *et al*., 2016a). Optimization of methods and support materials are important for cell immobilization; for example, the geometrical structure and surface chemistry of the support carriers directly influence microbial adhesion (Beshay and Moreira, 2003; Kourkoutas *et al*., 2005; Teughels *et al*., 2006; Dolejš *et al*., 2014). Therefore, it is necessary to choose an appropriate support material in order to establish an efficient immobilization system (Daniels *et al*., 2010; Zhang *et al*., 2015). Carriers should meet the following criteria: have a large surface, with functional groups for cells to adhere to; maintain the physical, chemical and biological stability; not affect cell growth, physiology or metabolic activity; and be inexpensive and simple to obtain, handle and regenerate (Yen and Li, 2011; Survase *et al*., 2013). Moreover, methods for immobilization should be easy to carry out, suitable for scale‐up, not affect cell activity, and maintain a strong connection between cells and carriers (Leenen *et al*., 1996; Willaert and Baron, 1996).

Cell attachment or adsorption can generally be carried out using simple methods with the advantages of low diffusion problems and at low cost (Kilonzo *et al*., 2011; Yao *et al*., 2011). The connection between the cell and carrier is typically mediated by electrostatic forces or covalent binding (Dolejš *et al*., 2014; Habouzit *et al*., 2014; Choi *et al*., 2015; Zhuang *et al*., 2016a). In this type of immobilization, the carriers are usually cellulosic or inorganic materials and can be modified by polycations, chitosan or other chemicals to enhance adsorption ability (Kayastha and Srivastava, 2001; Mamatarkova *et al*., 2014; Zhuang *et al*., 2016b). Cellulosic materials are easy to obtain, maintain high mechanical stability and have a high surface area rich in hydroxyl groups for modification (Baiardo *et al*., 2002; Belgacem and Gandini, 2005; Dankovich and Hsieh, 2007; de Oliveira Taipina *et al*., 2012; Tarbuk *et al*., 2014). Cell surface properties are the basis of material modification (Wilson *et al*., 2001; Holle *et al*., 2012; Choi *et al*., 2015).

In this study, we evaluated the roughness and specific surface properties of natural fibres (cotton, linen, bamboo fibre and silk) as carriers for immobilized butanol fermentation to determine the most suitable immobilization carrier for biomass formation and fermentation performance. We also examined the characteristics of *C. acetobutylicum* CGMCC 5234 to make corresponding surface modifications of the material for enhancing cell adsorption and fermentation performance.

## Results and discussion

### Carrier characterization

The structure and surface properties of carrier materials can influence the cell adsorption, biofilm formation and fermentation activity of immobilized microbes (Kourkoutas *et al*., 2004; Dolejš *et al*., 2014). Techniques for development of suitable immobilization carriers to achieve microbial cell immobilization have been explored. In previous studies, reducing the size of the material has been shown to promote the increase in activity of the immobilized biofilm due to changes in specific surface area, curvature‐induced associations and spatial confinement (Bucko *et al*., 2012).

In addition to surface chemical groups, topography and regularity have been shown to be associated with biofilm conformation (Leenen *et al*., 1996; Mamatarkova *et al*., 2014). These features can be attributed to enthalpic gains associated with polymer mobility and to weak interactions between the microbial and alternating surface that limit these enthalpic gains. Fibres with an irregular surface having high roughness and enriched in hydroxyl groups have generally been used to study immobilization of *C. acetobutylicum* (Dolejš *et al*., 2014). Therefore, in this study, we evaluated four natural fibres as potential carriers for immobilized fermentation.

**Figure 1:**
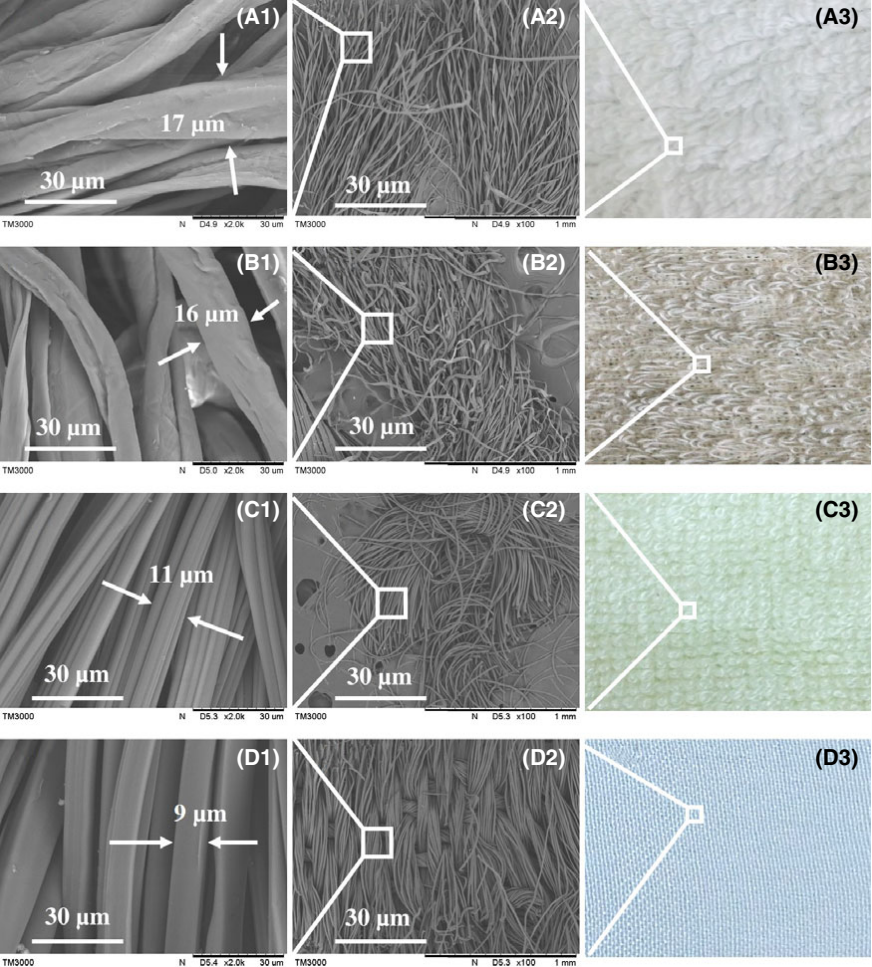
Images of the four potential carriers at different magnifications. The carriers were cotton (A), linen (B), bamboo fibre (C) and silk (D). The middle panels are enlarged images of the boxes in the right panels, and the left panels are enlarged images of the boxes in the middle panels.

Figure 1 shows the surface topographies of different carriers. Cotton and linen were about 16 μm wide, whereas bamboo fibre was 11 μm wide, and silk was 9 μm wide. Additionally, cotton and linen were rougher than silk and bamboo fibre. All four types of fibres were made of fibre bundles; cotton and linen fibres exhibited a similar density and were more densely than bamboo fibre, whereas silk exhibited the lowest density. The macroscopic appearances of fibres revealed that cotton, linen and bamboo fibres were composed of fibre bundles and that individual fibres were sometimes partly detached from the fibre bundle. In contrast, silk fibres exhibited a smooth surface. Thus, we concluded that cotton and linen fibres were more dense and rough, providing a higher specific surface area that contains more adsorption sites and adhesion force for bacterial cell adsorption.

### Fermentation performance

**Figure 2:**
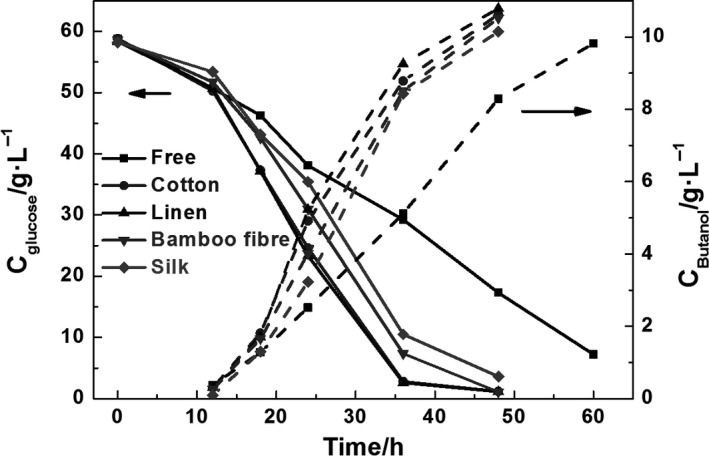
The kinetics of glucose consumption (solid lines and closed symbols) and butanol production (dashed lines and closed symbols) during free and immobilized fermentation with different support carrier materials.

Figure 2 shows the kinetics of glucose consumption and butanol production in the suspended and immobilized fermentations with different fibre carriers. Based on the fermentation results, we observed that the initial concentration of butanol was very low because the metabolic processes were focused on promoting bacterial growth. In the free fermentation, fermentation was complete in about 60 h, whereas in immobilized fermentation, fermentation was complete in about 48 h.

In addition, the rates of glucose consumption and butanol production were much slower in the free fermentation than in the immobilized fermentation. A comparison among immobilized fermentation with different carriers showed that the fermentation rates were similar for linen and cotton (requiring about 36 h to exhaust glucose) and that these values were slightly higher than those of the other carriers (requiring about 48 h to exhaust glucose).

**Figure 3:**
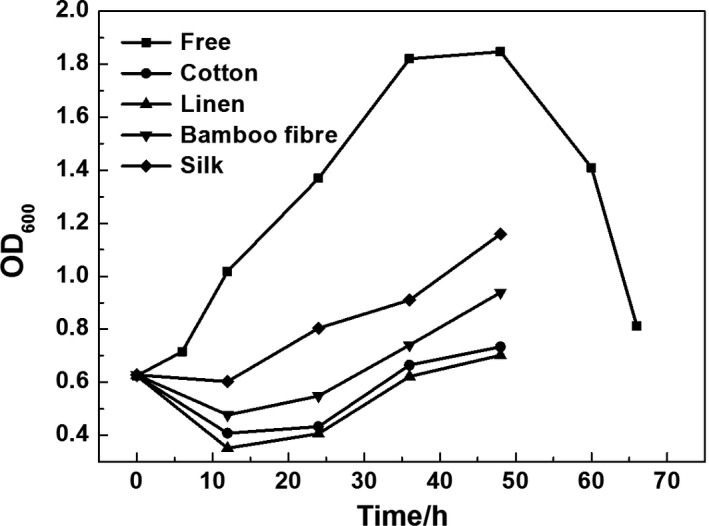
The density of bacterial cells in fermented liquid for free and immobilized fermentation with different support materials.

Figure 3 shows the density of bacteria in the fermented liquid from the immobilized and free fermentation. The density of the suspended cells was lower for immobilized fermentation than for free fermentation, suggesting that more cells had adsorbed to the carriers. During the later phases of fermentation, the suspended cells entered the decline phase of growth, and the bacterial cell density decreased. In contrast, in immobilized fermentation, biofilm cells could diffuse into the liquid, so no obvious decline was observed.

During early fermentation, the bacterial absorption rate was slower than the bacterial growth rate; decreased OD values represented the difficulty of bacterial adsorption to the surface. Thus, from our findings, we showed that bacteria could more easily be adsorbed onto linen. Moreover, cell density in fermented liquid corresponded to the fermentation performance; lower bacterial density in the liquid was associated with increased bacterial cells on carriers and improved fermentation performance (Kourkoutas *et al*., 2004; Dolejš *et al*., 2014).

Actually, it is shown that there was an increase in the OD during the fermentation, on the one hand, due to the higher growth rate compared with the adsorption rate. On the other hand, this increase in OD can be also due to the desorption of cells or to free cells in the culture special in the case of the silk carrier.

**Figure 4:**
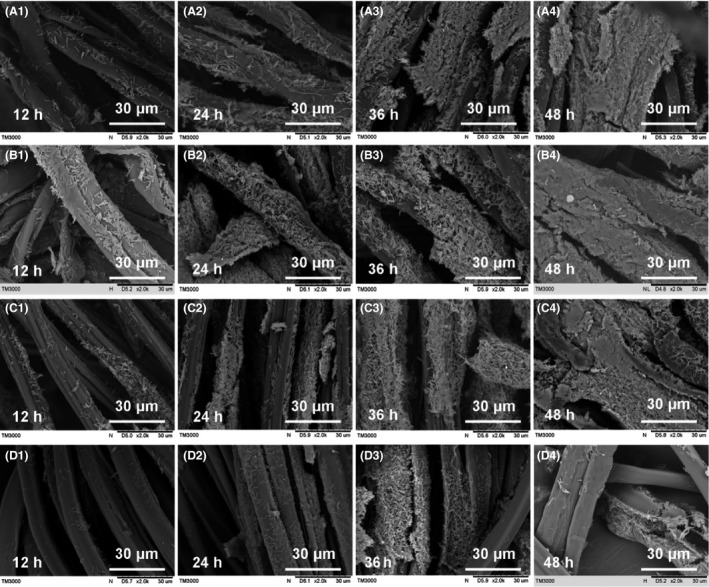
Scanning electron microscopy images of *Clostridium acetobutylicum* CGMCC 5234 cultured at different fermentation stages with different support carriers. Cotton (A), linen (B), bamboo fibre (C) and silk (D).

Next, we aimed to investigate the microscale mechanisms of immobilized fermentation by characterization of the biofilm formation process. Figure 4 shows the biofilm formation process on different carriers. In the initial adhesion phase, there were no obvious differences among cells immobilized on the four types of carriers. However, more cells were observed over time on cotton and linen owing to the improved adhesion ability on this material, consistent with the results of changes in liquid bacteria density. After bacterial proliferation, cell adsorption onto the carriers was increased, eventually forming mature biofilms. Biofilm on silk could be easily removed owing to the smooth surface of the material.

Proteins allow the developing biofilm to adhere to support surfaces; this process is thought to be associated with the roughness of the surface, with increased adherence of protein as the roughness increases (Gallardo‐Moreno *et al*., 2002; Goulter *et al*., 2010; Choi *et al*., 2015). In this study, our results of the analysis of material surface properties, bacteria adsorbed onto carriers and fermentation performance indicated that during immobilized fermentation, more bacteria were capable of adhering to rough materials for biofilm formation, thereby increasing bacterial density and improving fermentation rates.

As shown in Fig. 4 (d_4_), the biofilm could detach from the carrier. Thus, to examine the stability of immobilized fermentation, we evaluated the long‐term performance of immobilized *C. acetobutylicum* by repeated‐batch fermentations for biofilms formed on different carriers.

**Figure 5:**
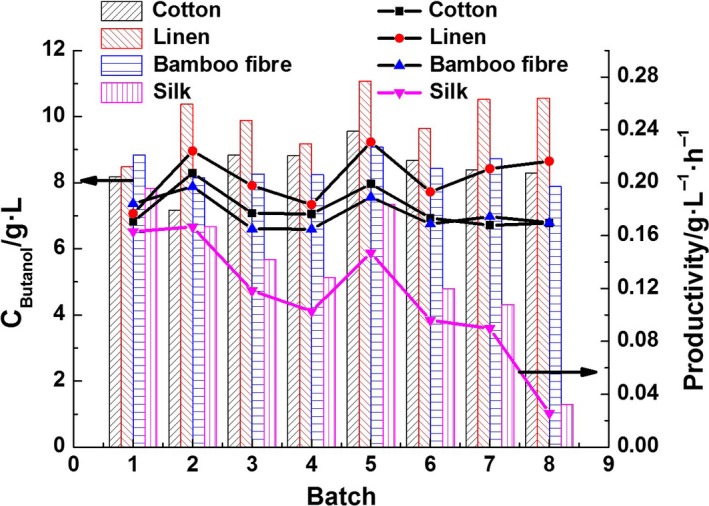
Butanol concentration and productivity in fed‐batch fermentation with different support carriers.

Figure 5 shows a comparison of immobilized fermentation for different carriers; these data indicated that *C. acetobutylicum* immobilized on linen converted glucose to butanol more efficiently and yielded higher average butanol concentrations (9.97 g l^−1^ versus 8.49 g l^−1^ for cotton, 8.45 g l^−1^ for bamboo fibre and 5.38 g l^−1^ for silk) and productivities (0.209 g l^−1^ h^−1^ versus 0.178 g l^−1^ h^−1^ for cotton, 0.177 g l^−1^ h^−1^ for bamboo fibre and 0.114 g l^−1^ h^−1^ for silk). From the results, we also found that the immobilization reaction system was stable when the carriers were cotton, linen and bamboo. After five batches, the fermentation performance with silk as the carrier was decreased as the biofilm was not firmly adhered to the material. Therefore, linen was found to be the most suitable carrier.

### Bacterial surface properties

Bacterial surface properties also affect adsorption and biofilm formation because material modification is often easier, cheaper and less detrimental than microbial modification (Choi *et al*., 2015; Luo *et al*., 2015). To enhance bacterial adsorption to carriers, we detected bacterial surface properties and evaluated appropriate modifications of the materials.

Surface hydrophobicity differs among organisms and is influenced by bacterial age, growth medium and bacterial surface structures (Kim *et al*., 2004; Liu *et al*., 2013). Therefore, we then measured bacterial surface hydrophobicity during different growth phases from fermentation medium with the BATH method (Table 1). Cells showed greater affinity for aqueous solution, indicating low levels of cell hydrophobicity for *C. acetobutylicum* CGMCC 5234.

**Table 1:** Affinities of *Clostridium acetobutylicum* CGMCC 5234 for hexadecane during various growth phases

Growth phase	Logarithmic phase	Stationary phase	Decline phase
*A*_0_	0.527	0.896	0.878
*A*_1_	0.377	0.594	0.768
1–*A*_1_/*A*_0_	0.2846	0.3371	0.1253

*A*_0_: cells suspended in PBS (0.1 M, pH 6.0).

*A*_1_: after mixing, cells in water.

The hydrophobic amino acid residues of fimbriae are the main factors affecting bacterial surface hydrophobicity. Previous studies have found a positive correlation between bacterial cell hydrophobicity and attachment to hydrophobic surfaces (Gallardo‐Moreno *et al*., 2002; Choi *et al*., 2015). Thus, although *C. acetobutylicum* CGMCC 5234 shows low hydrophobicity, it also plays a role in attachment of bacterial cells to abiotic surfaces.

The charge of the bacterial surface and carriers may lead to attraction or repulsion between bacteria and carriers; increasing the attraction may enhance the adsorption of cells to carriers (Buschle‐Diller *et al*., 2005). Because the charge is influenced by the pH of the solution, the pH of the fermented liquid was varied from 3.0 to 7.0 during the fermentation process (Wang *et al*., 2011). We then detected the cell surface charge using zeta potential measurements. As shown in Table 2, during the fermentation process, *C. acetobutylicum* exhibited a negative surface charge.

**Table 2:** Zeta potential of *Clostridium acetobutylicum CGMCC 5234* in PBS with different pH

pH	2.8	4.086	5.204	5.99	7.14
Zeta potential	−5.85	−21.3	−20	−25.03	−18.72

### Linen modification

#### Linen surface properties and bacterial adsorption after PEI and SA modifications

From our data presented above, we chose linen as the most suitable material for modification. Moreover, we found that during fermentation, *C. acetobutylicum* showed high hydrophilicity and a negative surface charge. Because the linen surface was rich in hydroxyl groups, we used PEI and SA to increase the positive charge and hydrophobicity, respectively, of the linen surface.

**Figure 6:**
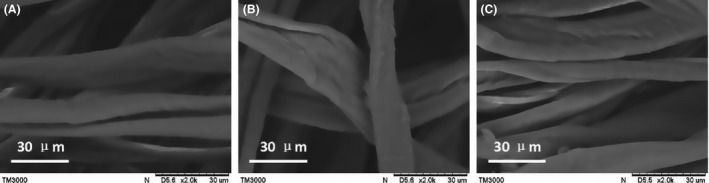
Scanning electron microscopy images of linen carrier fibres. (A) Linen, (B) PEI‐modified linen and (C) SA‐modified linen.

Figure 6 shows the surface of linen before and after modification. The Scanning electron microscopy (SEM) images showed that linen was rougher after modification, which may facilitate bacterial cell adsorption.

**Figure 7:**
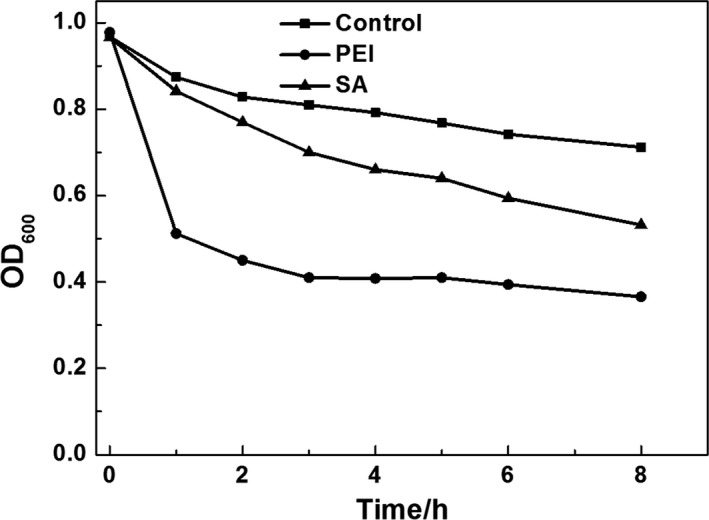
Densities of bacterial cells in PBS solution with modified and unmodified linen as immobilization carriers.

Figure 7 shows *C. acetobutylicum* static adsorption to linen carriers. From the cell density curves in solution, we found that linen modified by PEI reached adsorption equilibrium after about 2.5 h, with approximately 60% of the cells being adsorbed. Linen modified by SA adsorbed about 55% of cells after 8 h, while linen without modification could only adsorb about 28% of cells. Thus, we concluded that modified linen could increase bacterial adsorption capacity and adsorption rates. Moreover, the adsorption capacity of modified linen was about two times higher than that of unmodified linen.

#### Comparison of fermentation performance with modified and unmodified linen

**Figure 8:**
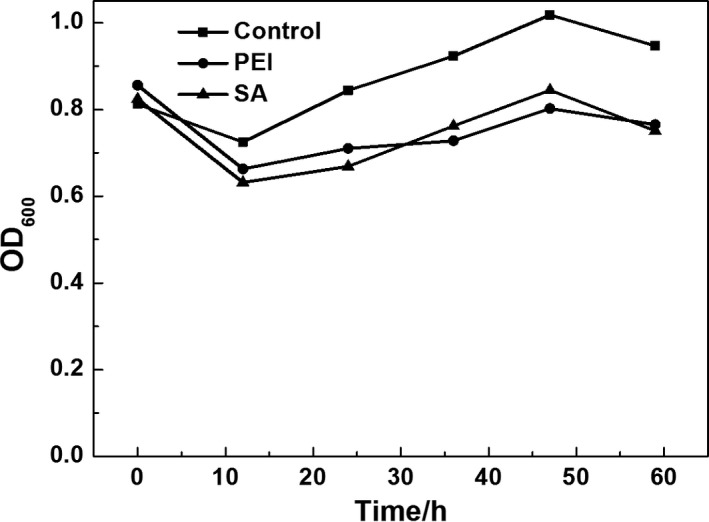
Density of bacterial cells in fermented liquid with unmodified linen and linen modified by PEI and SA.

Next, we examined the adherence and fermentation performance of bacterial cells in the presence of modified and unmodified linen as support material. As shown in Fig. 8, the density of bacterial cells in solution was decreased for modified linen compared with that of unmodified linen. These findings suggested that modification of the linen increased adsorption of cells to the modified carrier during fermentation; this may promote fermentation performance.

**Scheme 1:**
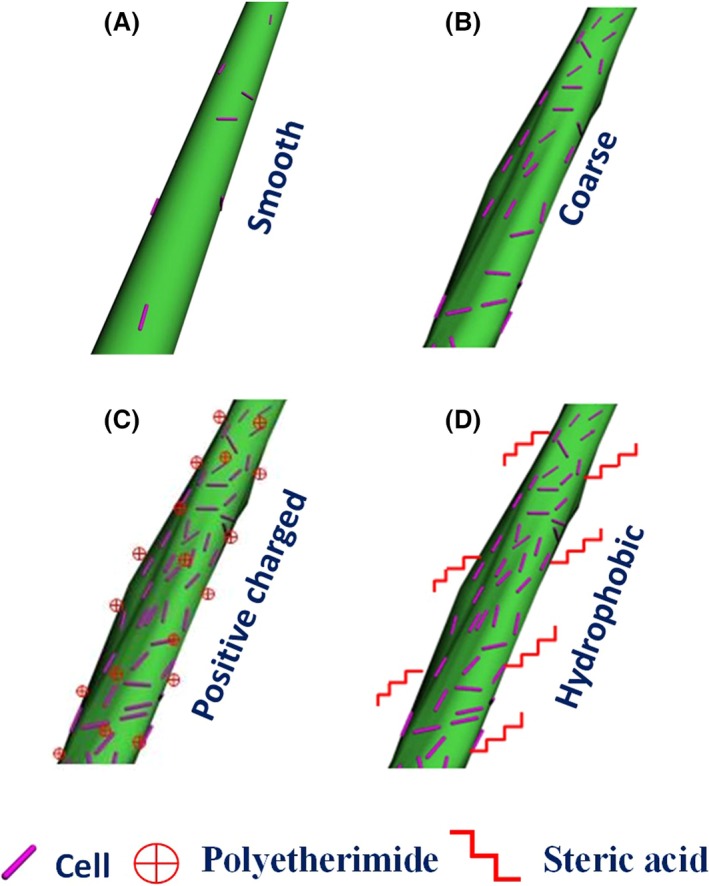
The effects of the structure and surface properties of natural textiles on cell adhesion: (A) Smooth structure of natural textiles such as silk with few cells adhered; (B) Coarse structure of natural textiles such as linen with a few cells adhered; (C) Coarse surface modified with positive charges leading to superior cell adhesion; (D) Coarse surfaces modified with hydrophobic chemicals with superior cell adhesion.

The main reason for the excellent performances is the effects of roughness and surface charge of different textiles on the adsorption of cells. As shown in Scheme 1(b), the cell adsorption performance of coarse surface structure of natural textiles is better than smooth one which is shown in Scheme 1(a). As described in previous work, the surface of both *C. acetobutylicum* and natural textiles is covered by negative charges, so the textiles modified with positive charge can enhance the adhesion of cells (Buschle‐Diller *et al*., 2005). In addition, another surface property of hydrophobicity can lead to the high efficient adsorption, superior biofilm formation and fermentation performances.

**Figure 9:**
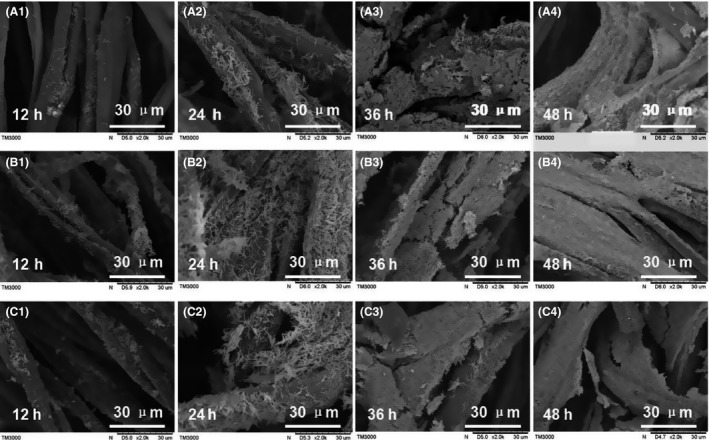
Scanning electron microscopy images of *Clostridium acetobutylicum* CGMCC 5234 cultured at different fermentation stages with different support carriers. (A) Linen, (B) PEI‐modified linen and (C) SA‐modified linen.

Further microscopic observations of the biofilm formation process on modified and unmodified linen are shown in Fig. 9. In the initial adhesion phase, more bacteria were adhered to the modified linen than to the unmodified linen; however, after 36 h, there were no apparent differences in biofilm formation among the different support carriers. Based on combined macroscopic and microscopic images, we concluded that after modification, the linen carrier could adsorb more bacteria during the fermentation process.

Table 3 shows the results of immobilized fermentation with modified and unmodified linen as support carriers. We found that fermentation performance was slightly increased when modified linen was used as the carrier, although part of the performance is not superior to previous literatures. After modification by PEI and SA, the butanol productivity and yield were increased by 8.16% and 6.80% and by 15.00% and 5.00%, respectively, compared with samples in which unmodified linen was used as a carrier.

**Table 3:** Comparison of butanol concentrations, productivity and yield during immobilized fermentation using modified and unmodified linen as a support carrier

Carrier	Ethanol (g l^−1^)	Acetone (g l^−1^)	Acetoin (g l^−1^)	Butanol (g l^−1^)	Acetic acid (g l^−1^)	Butyric acid (g l^−1^)	Productivity of ABE (g l^−1^ h^−1^)	Yield of ABE (g g^−1^)	Reference
Linen	1.56 ± 0.06	4.62 ± 0.13	1.18 ± 0.04	9.56 ± 0.54	0.72 ± 0.03	0.34 ± 0.02	0.35	0.36	This work
Linen‐PEI	1.46 ± 0.04	5.22 ± 0.15	1.32 ± 0.05	10.34 ± 0.43	0.79 ± 0.05	0.32 ± 0.02	0.38	0.38	This work
Linen‐SA	1.52 ± 0.06	4.83 ± 0.13	1.26 ± 0.04	10.21 ± 0.52	0.88 ± 0.03	0.37 ± 0.03	0.37	0.36	This work
Hydrogel	–	0.41	–	2.23	–	–	0.49	0.15	Dolejš *et al*. (2014)
Wood pulp fibres	–	–	–	–	–	–	5.22	0.30	Survase *et al*. (2013)
Bricks	–	–	–	9.94 ± 1.66	–	–	0.47	0.22	Yen and Li (2011)
Fibrous matrix	1.6	6.1	1.0	11.5	0.86	0.45	0.37	0.34	Liu *et al*. (2015)

## Conclusions

In summary, in this study, we found that linen, which had a higher specific surface area and roughness, could adsorb more bacteria during immobilized fermentation, resulting in improved fermentation performance. In fed‐batch fermentation, the average butanol productivity with linen as a carrier was higher than those of cotton, bamboo fibre and silk. We also found that during fermentation, cells showed high hydrophilicity and a negative charge; therefore, we modified linen with PEI to increase cations on the linen surface and with SA to increase the hydrophobicity of the linen surface. These modifications increased cell adhesion and butanol production, supporting the use of modified linen as a carrier material in immobilized fermentation.

## Experimental procedures

### Growth medium and *C. acetobutylicum*

*Clostridium acetobutylicum* CGMCC 5234 was preserved in our laboratory and stored in 30% (v/v) glycerol at –80°C. The modified P2 medium, which contained 3 g l^−1^ yeast extract, 5 g l^−1^ peptone, 10 g l^−1^ glucose, 2 g l^−1^ CH_3_COONH_4_, 2 g l^−1^ NaCl, 3 g l^−1^ MgSO_4_·7H_2_O, 1 g l^−1^ KH_2_PO_4_, 1 g l^−1^ K_2_HPO_4_ and 0.1 g l^−1^ FeSO_4_·7H_2_O, was used for cell propagation. Solid medium was prepared by addition of 15–30 g l^−1^ agar for activation of the bacterial strain. The production medium contained 60 g l^−1^ glucose, 0.5 g l^−1^ K_2_HPO_4_, 0.5 g l^−1^ KH_2_PO_4_, 2.2 g l^−1^ CH_3_COONH_4_, 0.2 g l^−1^ MgSO_4_·7H_2_O, 0.01 g l^−1^ MnSO_4_·H_2_O, 0.01 g l^−1^ NaCl, 0.01 g l^−1^ FeSO_4_·7H_2_O, 1 mg l^−1^ p‐aminobenzoic acid, 1 mg l^−1^ thiamine and 0.01 mg l^−1^ biotin (Chen *et al*., 2013; Liu *et al*., 2014).

### Carrier materials

Cotton, linen, bamboo fibre and silk were purchased from a local market. The samples were cut into small squares (6 × 6 cm^2^), washed with distilled water and dried at 60°C before use. Linen was modified by polyetherimide (PEI) and steric acid (SA, Aladdin, Shanghai, China). The carriers were pretreated by soaking in 4% NaOH, boiling for 1 h, washing with deionized water to neutral and drying at 60°C (Belgacem and Gandini, 2005; de Oliveira Taipina *et al*., 2012).

Polyetherimide modification was carried out as follows: first, the pretreated materials were soaked in 50% PEI aqueous solution (adjusted to pH 7.0 by HCl) for 2 h at room temperature. Materials were then washed twice with deionized water and dried at 60°C (Tarbuk *et al*., 2014).

Steric acid modification was carried out as follows: first, the pretreated materials were soaked in absolute ethyl alcohol with 2% (w/w) SA for 2 h at room temperature. Samples were then dried at 105°C for 45 min, washed with absolute ethyl alcohol and air‐dried (Dankovich and Hsieh, 2007).

### Cell surface characteristics

Bacterial adhesion to solvents (BATS) assays were used for detection of hydrophobicity (Rosenberg *et al*., 1980). Bacteria were harvested at the logarithmic, stationary or declining phase by centrifugation (8000 rpm, 10 min, 4°C), then washed twice with potassium phosphate buffer (PBS; 0.1 M, pH 6.0) and resuspended in PBS. The bacterial suspensions were adjusted to an optical density (OD) of 0.8–1.0 at 400 nm (*A*_0_). Next, 4.8 ml of the bacterial suspensions and 0.8 ml of n‐hexadecane were vortexed for 90 s. After allowing the mixtures to stand for 15 min to allow the solvent phase to rise completely, the aqueous phase was carefully removed with a pipette for measurement at 400 nm using a spectrophotometer (*A*_1_). The ratio of *A*_1_ to *A*_0_ was calculated as a percentage of cells bound to the solvent using the following equation:

$$\text{Hydrophobicity}\;\left(\%\right)=\frac{A_{0}-A_{1}}{A_{0}}\times 100\%.$$

Zeta potential assays were used to detect cell surface charge (Wilson *et al*., 2001). Bacteria were harvested by centrifugation (8000 rpm, 10 min, 4°C), washed twice with deionized water and resuspended in solutions of pH 3.0, 4.0, 5.0, 6.0 or 7.0 adjusted by 0.1 M NaOH or 0.1 M HNO_3_.

### Bacteria immobilization

Vegetative cells were harvested by centrifugation (8000 rpm, 10 min and at 4°C), washed twice with PBS (0.1 M, pH 6.0) and resuspended in PBS to an OD of 0.7–0.9 at 600 nm. Next, 100 ml of the bacteria solution containing 6 × 6 cm^2^ carrier textiles free in the medium was placed in screw‐capped bottles (250 ml) and incubated at 25°C with shaking in thermostatic air table at 120 rpm to induce natural cell adhesion. Measurements were carried out at an OD of 600 nm (OD_600_) every hour until the OD value plateaued. Each measurement of the immobilized fermentation was conducted in triplicate; the plotted data represent the average value.

### Batch and fed‐batch fermentation

Batch fermentation with *C. acetobutylicum* CGMCC 5234 cells was carried out in screw‐capped bottles (250 ml) in an anaerobic chamber at 37°C, and 100 ml of the production medium was inoculated with 10 ml of the vegetative cell suspension. The immobilized fermentation was then carried out with support carriers in this medium.

Fed‐batch fermentation with immobilized cells was also carried out in screw‐capped bottles (250 ml) in thermostatic air table. All experiments were carried out in triplicate. When the concentration of glucose fell to less than 10 g l^−1^, the old broth was removed and 100 ml fresh broth was added (Chen *et al*., 2013). The yield of ABE was calculated as the total solvent (ABE) produced (g) divided by the glucose consumed (g). Initial glucose formula was 60 g l^−1^.

### Analytical methods

Cell density was analysed by measuring the OD_600_ with a spectrophotometer (Model 7200; Unico Instrument, Shanghai, China). Acetone, ethanol, butanol and acetic acid were determined by gas chromatography (model 7890A; Agilent Technologies, Santa Clara, CA, USA) equipped with a flame ionization detector, an autosampler (model 7693; Agilent Technologies) and a glass column (HP‐INNOWax, 60 m × 0.25 mm × 0.5 μm; Agilent Technologies). The oven temperature was programmed to increase from 70 to 190°C at a gradient of 20°C min^−1^, with an initial holding time of 0.5 min and a post‐holding time of 4 min. The injector and detector temperatures were maintained at 180 and 220°C respectively. Samples were first passed through a 0.22‐μm syringe filter with an injection volume of 1 μl. Nitrogen was used as a carrier gas at a flow rate of 30 ml min^−1^ (Chen *et al*., 2013; Liu *et al*., 2014).

Glucose was measured at OD_540_ with a spectrophotometer, and 0.5 ml of the sample and 0.5 ml DNS (mainly containing 3,5‐dinitrosalicylic acid, phenol and seignette salt) were mixed together, boiled for 5 min, then cooled in ice water immediately. Deionized water (8 ml) was then added for detection (Chen *et al*., 2013).

### Scanning electron microscopy

Cell‐immobilized support carriers were fixed in 2.5% glutaraldehyde in 0.2 M PBS (pH 7.3–7.4) for over 4 h and washed twice with deionized water. Cells were fixed again using 1% osmium tetroxide in 0.1 M PBS (pH 7.0–7.5) for 1–2 h and washed twice with deionized water, followed by dehydration in a graded ethanol series. After treatment, the samples were sputter‐coated with gold–palladium for 2 min in a Polaron E5100 Series II sputter coater (Polaron Equipment, Watford, Hertfordshire, UK), and specimens were viewed by SEM at an accelerating voltage of 10 kV (Obana *et al*., 2014; Semenyuk *et al*., 2014).

## Conflict of interest

None declared.
